# Donor History of Drug Use and Graft Survival in Pediatric Heart Transplant Recipients

**DOI:** 10.1001/jamanetworkopen.2025.7766

**Published:** 2025-04-28

**Authors:** Paul Esteso, Kimberlee Gauvreau, Caitlin Milligan, Linda Vo, Francis Fynn-Thompson, Elizabeth D. Blume, Tajinder P. Singh

**Affiliations:** 1 Department of Cardiology, Boston Children’s Hospital, Boston, Massachusetts; 2 Department of Pediatrics, Harvard Medical School, Boston, Massachusetts; 3 Department of Biostatistics, Harvard School of Public Health, Boston, Massachusetts; 4 Department of Cardiothoracic Surgery, Boston Children’s Hospital, Boston, Massachusetts

## Abstract

**Importance:**

Older children awaiting a heart transplant (HT) sometimes receive a heart offer from a donor with a history of drug use (HDU). The effect of using such donor hearts on posttransplant survival in pediatric recipients is unclear.

**Objective:**

To assess the association of using hearts from donors with HDU on posttransplant graft survival in pediatric HT recipients.

**Design, Setting, and Participants:**

For this retrospective cohort study, all pediatric HT recipients (aged <18 years) in the Organ Procurement and Transplantation Network database during January 1, 2000, to December 31, 2020, were identified. Among the recipients who received a heart from a donor with HDU, nearly all donors were aged 11 years or older. A propensity score (PS) model was developed to assess the probability of receiving a heart from a donor with HDU using baseline recipient and donor variables, limiting the study cohort to donors aged 11 years or older. Data were analyzed from October 2023 to November 2024.

**Exposure:**

HT using a heart from a donor with HDU (exposure group) vs from a donor without HDU (control group).

**Main Outcomes and Measures:**

The main outcome was graft loss (death or retransplant) assessed at 90 days after transplant and long term among 90-day survivors. Kaplan-Meier survival curves and a Cox proportional hazards regression model that accounted for matching of exposure and control groups were used to compare risk of graft loss.

**Results:**

This study included 2730 pediatric HT recipients. Their median age at HT was 14 years (IQR, 11-16 years), and most (1642 [60.1%]) were male. Overall, the exposure group comprised 822 children who received a heart from a donor with HDU; of these, 765 (93.1%) were PS matched to the control group. There was no difference in risk of graft loss within 90 days (hazard ratio [HR], 0.93 [95% CI, 0.55-1.57]; *P* = .78) or at long-term follow-up (HR, 1.04 [95% CI, 0.87-1.25]; *P* = .68) between PS-matched groups. Risk of graft loss within 90 days was not significantly different in children who received a heart from a donor with a history of cocaine use (157 pairs) vs children in the control group (HR, 0.55 [95% CI, 0.19-1.54]; *P* = .25); however, the risk of long-term graft loss among 90-day survivors was significantly higher (HR, 2.03 [95% CI, 1.35-3.06]; *P* = .001).

**Conclusions and Relevance:**

In this cohort study of pediatric HT recipients, there was no association of 90-day graft survival with donor HDU; however, donor history of cocaine use was associated with a higher risk of long-term graft loss. These findings may be important when considering a donor with HDU for pediatric HT candidates.

## Key Points

**Question:**

What is the association of donor history of drug use (HDU) with posttransplant graft loss (death or retransplant) in pediatric heart transplant (HT) recipients?

**Findings:**

This cohort study involved 2730 children younger than 18 years who received a HT between 2000 and 2020, with follow-up until 2022. There was no association of 90-day graft loss with receipt of a heart from a donor with HDU; however, donor cocaine use was associated with higher risk of long-term graft loss.

**Meaning:**

These findings suggest that donor history of cocaine use may be important to consider when assessing donors with HDU for pediatric HT candidates.

## Teaser

This cohort study evaluates the association of donor history of drug use with posttransplant graft loss among pediatric heart transplant recipients.

## Introduction

Heart transplantation in children with end-stage heart failure is constrained by the limited availability of donor organs as the number of children listed for a heart transplant (HT) continues to rise.^1^ One potential source of donors for older children in the US may be young donors with a history of drug use (HDU), who, if lacking additional risk factors, may be reasonable to consider. There has been a notable increase in recent years in the proportion of donors with HDU in the US, owing to the opioid epidemic.^2,3^ Studies in adult HT recipients have found no association of donor HDU with posttransplant survival.^2,3,4^ Pediatric data on graft survival using hearts from donors with HDU are limited, and none have accounted for recipient and donor risk factors.^5^ With median graft survival approaching 20 years, pediatric HT recipients have decades of life ahead of them and, unlike in adults, the onset of graft failure leads to a discussion of a second HT rather than end-of-life care.^1,6,7^ Understanding the effect of donor factors such as HDU on long-term graft survival may therefore be far more important in children.

The purpose of this study was to assess the association of using hearts from donors with HDU with posttransplant graft survival in pediatric HT recipients. Because the distribution of risk factors in children receiving a heart from a donor with HDU may be systematically different from children who receive a heart from a donor with no HDU, we assessed this association using a propensity score (PS)–matched analysis^8^ to compare posttransplant graft survival (freedom from death or retransplant) between groups from 2000 to 2020.

## Methods

### Study Setting and Participants

For this retrospective cohort study, we identified all children aged younger than 18 years in the Organ Procurement and Transplantation Network (OPTN) database who received a primary HT in the US between January 1, 2000, and December 31, 2020. The OPTN database includes baseline and follow-up data, including patient death, for all recipients in the US submitted by transplant centers. The death data are supplemented by the Social Security Administration Death Master File. These data are available deidentified from the United Network for Organ Sharing (UNOS) for a nominal fee. The study was deemed not human participant research by the Boston Children’s Hospital Institutional Review Board; thus, informed consent was waived. The study followed the Strengthening the Reporting of Observational Studies in Epidemiology (STROBE) reporting guideline.

An initial assessment showed that of 838 patients aged younger than 18 who received a heart from a donor with HDU, the donor age was 11 years or older for most (822 [98.1%]). As a first step in making the distribution of baseline characteristics between the exposure and control groups comparable, we limited the study cohort to children who received a heart from a donor aged older than 11 years. Posttransplant follow-up was available for all patients until March 31, 2022.

### Study Design and Variables

The primary outcome of interest was graft loss (death or retransplant) assessed using time-to-event analysis. This outcome was analyzed for the first 90 days post transplant and long term in 90-day survivors. The exposure was donor HDU, and outcomes were compared between pediatric patients who received a heart from a donor with HDU (exposure group) and PS-matched pediatric patients who received a heart from a donor with no HDU (control group).

Baseline variables were defined at transplantation. Donor HDU is defined in the OPTN data as the history of the donor ever using or having a dependence on cocaine or on other drugs (nonintravenous street drugs such as crack, marijuana, or prescription narcotics, sedatives, hypnotics, or stimulants).^5^ We did not consider donor alcohol use. Patient race and ethnicity was assessed due to the known association of Black race with posttransplant outcomes. Race and ethnicity is a mandatory variable in OPTN data and is submitted by the transplant center as American Indian or Alaska Native, Asian, Black, Hispanic or Latino, Native Hawaiian or Other Pacific Islander, White, multiple races or ethnicities, or other race or ethnicity. Due to the small sample sizes in racial and ethnic groups other than Black, Hispanic, and White, we categorized HT recipients in 4 groups: Black (non-Hispanic), Hispanic, White (non-Hispanic), or other race or ethnicity. Kidney function was analyzed as the estimated glomerular filtration rate (eGFR) (in milliliters per minute per 1.73 m^2^) using serum creatinine and the modified Schwartz equation.^9^ Normal kidney function was defined as an eGFR greater than 60, moderate dysfunction as an eGFR of 30 to 60, and severe dysfunction as an eGFR of less than 30 or receipt of dialysis support.

There were no missing data for variables age, sex, race and ethnicity, cardiac diagnosis, blood type, hemodynamic support (inotrope, ventilator, mechanical support), health insurance (Medicaid), dialysis, and the dates of transplant, death, or retransplant. For children with missing values for serum creatinine (23 [0.8%]) or bilirubin (97 [3.6%]), we used multiple imputation to impute their eGFR and serum bilirubin, respectively, using clinical variables at transplant; 10 imputations were used for each missing value.^10^

### Statistical Analysis

Patient characteristics are presented as medians (IQRs) or numbers with percentages. Baseline characteristics were compared between the exposure and control groups using the Wilcoxon rank-sum test for continuous variables and the Fisher exact test for categorical variables. To attain a balance of baseline characteristics between groups, a PS-matched analysis was performed. We developed a logistic regression model with the outcome of interest (receipt of a heart from a donor with HDU) using 10 recipient variables (age, sex, weight, diagnosis, race and ethnicity, ventilator, mechanical support, dialysis, eGFR, and serum bilirubin) and 6 donor variables (age, donor-recipient age difference, donor weight, donor-recipient weight ratio, donor left ventricular ejection fraction <0.5, and use of ≥2 inotropes). The model was used to generate a PS (probability of receiving a heart from a donor with HDU) for each HT recipient. Children in the exposure group were matched 1:1 with those in the control group using a nearest-neighbor algorithm (greedy matching), with a maximum PS caliper of 0.01. Standardized mean differences were calculated to assess balance between matched groups. Kaplan-Meier survival curves were used to compare survival between PS-matched groups. A Cox proportional hazards regression model with robust variance estimators that accounted for PS matching and a Cox proportional hazards regression model that additionally adjusted for recipient age, donor age, and year of transplant were used to assess the association of donor HDU with posttransplant graft loss. Schoenfeld residual plots were used to evaluate the assumption of proportional hazards. Similar analyses were performed to compare risk of graft loss in children who received a heart from a donor with a history of cocaine use to PS-matched children who received a heart from a donor with no HDU (control group). Kaplan-Meier curves were used to compare time to diagnosis of coronary artery vasculopathy, as reported by centers in annual follow-up reports, between children who received a heart from a donor with history of cocaine use and children in the control group.

Data were analyzed using SAS, version 9 (SAS Institute Inc), and Stata, version 17 (StataCorp). All tests were 2-sided, and *P* < .05 was considered significant. Data were analyzed from October 2023 to November 2024.

## Results

### Study Population

During the study period, 7290 children underwent a first HT in the US. Of these, 2730 children received a heart from a donor aged 11 years or older, and these children formed the study cohort. Their median age was 14 years (IQR, 11-16 years); 1088 (39.9%) were female and 1642 (60.1%) were male. The indication for HT was cardiomyopathy in 1861 children (68.2%) and congenital heart disease in 845 children (31.0%). There were 822 children (30.1%) in the exposure group and 1908 children (69.9%) in the control group. Baseline characteristics between groups are compared in Table 1. Children in the exposure group were older at the time of HT, were more likely to be receiving mechanical circulatory support, and had older donors. The percentage of donors with HDU increased from 19.3% (51 of 264) during 2000 to 2002 to 39.7% (229 of 577) during 2018 to 2020.

**Table 1.** Baseline Characteristics of Pediatric Heart Transplant Recipients by Donor History of Drug Use, 2000-2020^a^

Characteristic	Total cohort (N = 2730)	Exposure group (n = 822)	Control group (n = 1908)	*P* value
**Patient**				
Age at transplant, y				
1-10	520 (19.1)	94 (11.4)	426 (22.3)	<.001
11-17	2210 (80.9)	728 (88.6)	1482 (77.7)	
Weight, kg	50 (10-146)	56 (15-146)	48 (10-137)	<.001
Sex				
Female	1088 (39.9)	313 (38.1)	775 (40.6)	.22
Male	1642 (60.1)	509 (61.9)	1133 (59.4)	
Diagnosis				
Dilated cardiomyopathy	1504 (55.1)	471 (57.3)	1033 (54.1)	.26
Nondilated cardiomyopathy	357 (13.1)	106 (12.9)	251 (13.2)	
CHD repaired	782 (28.6)	217 (26.4)	565 (29.6)	
CHD unrepaired	63 (2.3)	23 (2.8)	40 (2.1)	
Other	24 (0.9)	5 (0.6)	19 (1.0)	
Race and ethnicity				
Black	654 (24.0)	201 (24.5)	453 (23.7)	.67
Hispanic	470 (17.2)	144 (17.5)	326 (17.1)	
White	1438 (52.7)	421 (51.2)	1017 (53.3)	
Other^b^	168 (6.2)	56 (6.8)	112 (5.9)	
Ventilator	199 (7.3)	59 (7.2)	140 (7.3)	.94
Mechanical support				
ECMO	86 (3.2)	30 (3.7)	56 (2.9)	.01
BIVAD	183 (6.7)	60 (7.3)	123 (6.5)	
LVAD	526 (19.3)	185 (22.5)	341 (17.9)	
None of the above	1935 (70.9)	547 (66.6)	1388 (72.8)	
Prior cardiac surgery	946 (34.7)	255 (31.0)	691 (36.2)	.01
Dialysis	84 (3.1)	28 (3.4)	56 (2.9)	.55
Serum creatinine (n = 2709, 1890, and 819)	0.7 (0.04-23)	0.7 (0.04-17)	0.7 (0.1-23)	.01
eGFR (n = 2707, 1888, and 819)	94.4 (2.6-150)	93.1 (4.1-150)	95.5 (2.6-150)	.60
Bilirubin, mg/dL (n = 2633, 1828, and 805)	0.7 (0.1-41.0)	0.7 (0.1-34.0)	0.7 (0.1-41.0)	.56
Year of transplant				
2000-2002	264 (9.7)	51 (6.2)	213 (11.2)	<.001
2003-2005	320 (11.7)	83 (10.1)	237 (12.4)	
2006-2008	346 (12.7)	86 (10.5)	260 (13.6)	
2009-2011	359 (13.2)	105 (12.8)	254 (13.3)	
2012-2014	397 (14.5)	124 (15.1)	273 (14.3)	
2015-2017	467 (17.1)	144 (17.5)	323 (16.9)	
2018-2020	577 (21.1)	229 (27.9)	348 (18.2)	
**Donor**				
Donor age, y				
11-17	1595 (58.4)	289 (35.2)	1306 (68.5)	<.001
18-25	725 (26.6)	339 (41.2)	386 (20.2)	
26-35	315 (11.5)	160 (19.5)	155 (8.1)	
≥36	95 (3.5)	34 (4.1)	61 (3.2)	
Donor age minus patient age, y	4 (0-47)	6 (0-37)	4 (0-47)	<.001
Donor weight, kg (n = 2723, 1901, and 822)	63 (20-157)	68 (35-157)	60 (20-147)	<.001
Recipient:donor weight ratio (n = 2722, 1900, and 822)	0.81 (0.27-1.87)	0.82 (0.29-1.81)	0.81 (0.27-1.87)	.04
LVEF <50% (n = 2653, 1840, and 813)	43 (1.6)	7 (0.9)	36 (2.0)	.04
≥2 Inotropes used	246 (9.0)	55 (6.7)	191 (10.0)	.006

Abbreviations: BIVAD, biventricular assist device; CHD, congenital heart disease; ECMO, extracorporeal membrane oxygenation; eGFR, estimated glomerular filtration rate; LVAD, left ventricular assist device; LVEF, left ventricular ejection fraction.

^a^ Values are reported as the No. (%) of patients or median (range). The exposure group comprised children who received a heart from a donor with a history of drug use, whereas the control group comprised children who received a heart from a donor with no history of drug use.

^b^ Includes American Indian or Alaska Native, Asian, Native Hawaiian or Other Pacific Islander, and multiple races or ethnicities.

Table 2 compares baseline characteristics among 765 pairs of HT recipients matched for their probability of receiving a heart from a donor with HDU. These exposure and control groups were well matched for the distribution of all measured variables. The 57 children in the exposure group who could not be matched were older, and they were more likely to receive a heart from an older donor with a larger donor-recipient age difference. Among the 765 PS-matched children in the exposure group, 560 donors (73.2%) had used drugs within 6 months of HT. Furthermore, 157 children (20.5%) in the exposure group received a heart from a donor with a history of cocaine use.

**Table 2.** Baseline Characteristics of Pediatric Heart Transplant Recipients Propensity Matched for Donor History of Drug Use, 2000-2020^a^

Characteristic	Exposure group (n = 765)	Control group (n = 765)	Standardized mean difference
**Patient**			
Age at transplant, y			
1-10	93 (12.2)	70 (9.1)	0.10
11-17	672 (87.8)	695 (90.9)	
Weight, kg	55 (15-146)	55 (10-137)	0.01
Sex			
Female	290 (37.9)	299 (39.1)	−0.02
Male	475 (62.1)	466 (60.9)	
Diagnosis			
Dilated cardiomyopathy	441 (57.7)	447 (58.4)	−0.02
Nondilated cardiomyopathy	98 (12.8)	101 (13.2)	−0.01
CHD repaired	198 (25.9)	192 (25.1)	0.02
CHD unrepaired	23 (3.0)	20 (2.6)	0.02
Other	5 (0.7)	5 (0.7)	0
Race and ethnicity			
Black	186 (24.3)	193 (25.2)	−0.02
Hispanic	136 (17.8)	136 (17.8)	0
White	390 (51.0)	378 (49.4)	0.03
Other^b^	53 (6.9)	58 (7.6)	−0.02
Ventilator	56 (7.3)	54 (7.1)	0.01
Mechanical support			
ECMO	27 (3.5)	23 (3.0)	0.03
BIVAD	54 (7.1)	52 (6.8)	0.01
LVAD	168 (22.0)	169 (22.1)	−0.003
None of above	516 (67.5)	521 (68.1)	−0.01
Dialysis	26 (3.4)	23 (3.0)	0.02
Serum creatinine	0.7 (0.04-7)	0.7 (0.1-20)	0.02
eGFR	92.9 (10.5-150)	94.4 (3.8-150)	0.03
Bilirubin, mg/dL	0.7 (0.1-34)	0.8 (0.1-41)	0.02
**Donor**			
Donor age, y			
11-17	289 (37.8)	294 (38.4)	−0.01
18-25	304 (39.7)	302 (39.5)	0.01
26-35	140 (18.3)	135 (17.7)	0.02
≥36	32 (4.2)	34 (4.4)	−0.01
Donor age minus patient age, y	6 (0-37)	6 (0-47)	−0.01
Donor weight, kg	68 (35-157)	67 (28-147)	0.001
Recipient-donor weight ratio	0.82 (0.29-1.67)	0.84 (0.31-1.73)	0.06
LVEF <50%	7 (0.9)	3 (0.4)	0.06
≥2 Inotropes used	55 (7.2)	60 (7.8)	−0.02

Abbreviations: BIVAD, biventricular assist device; CHD, congenital heart disease; ECMO, extracorporeal membrane oxygenation; eGFR, estimated glomerular filtration rate; LVAD, left ventricular assist device; LVEF, left ventricular ejection fraction.

^a^ Values are reported as No. (%) of patients or median (range). The exposure group comprised children who received a heart from a donor with a history of drug use, whereas the control group comprised children who received a heart from a donor with no history of drug use.

^b^ Includes American Indian or Alaska Native, Asian, Native Hawaiian or Other Pacific Islander, and multiple races or ethnicities.

### Posttransplant 90-Day Graft Survival

**Figure 1.**
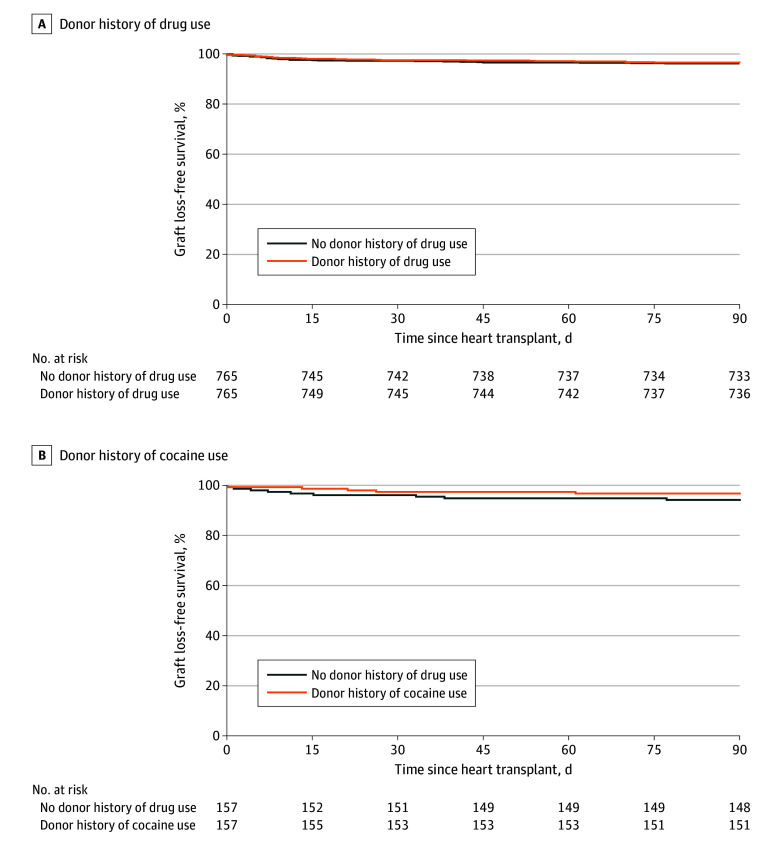
Ninety-Day Graft Survival in Pediatric Patients Who Received a Heart From a Donor With vs Without a History of Drug or Cocaine Use, 2000-2020

Of the 765 children in the exposure group, 27 (3.5% [25 deaths and 2 retransplants]) reached the primary end point within 90 days compared with 29 (3.8% [27 deaths and 2 retransplants]) in the control group. Figure 1A compares 90-day graft survival in the PS-matched exposure vs control groups. There was no significant difference in graft survival between groups. In a Cox proportional hazards regression model accounting for matching, there was no association of donor HDU with recipient risk of 90-day graft loss (hazard ratio [HR], 0.93 [95% CI, 0.55-1.57]; *P* = .78). These findings remained unchanged when the model was additionally adjusted for year of transplant, recipient age, and donor age (HR, 0.95 [95% CI, 0.56-1.62]; *P* = .85). Furthermore, in a Cox proportional hazards regression model limited to 560 PS-matched recipient pairs in which the donor had used drugs within 6 months before transplant, there was no association of donor HDU with recipient 90-day graft loss (HR, 1.12 [95% CI, 0.58-2.18]; *P* = .73) or when further adjusted for year of transplant, recipient age, and donor age (HR, 1.11 [95% CI, 0.572.17]; *P* = .75).

Of the 157 children who received a heart from a donor with a history of cocaine use, 5 (3.2% [5 deaths]) reached the primary end point by 90 days post transplant compared with 9 (5.7% [8 deaths and 1 retransplant]) in the PS-matched control group. Figure 1B compares 90-day graft survival between 157 children who received a heart from a donor with a history of cocaine use and 157 PS-matched children in the control group, showing no significant difference in graft survival. In a Cox proportional hazards regression model accounting for matching, there was no association of donor history of cocaine use with risk of graft loss within 90 days (HR, 0.55 [95% CI, 0.19-1.54]; *P* = .25) or when further adjusted for year of transplant, recipient age, and donor age (HR, 0.50 [95% CI, 0.17-1.44]; *P* = .21).

### Long-Term Graft Survival in 90-Day Survivors

**Figure 2.**
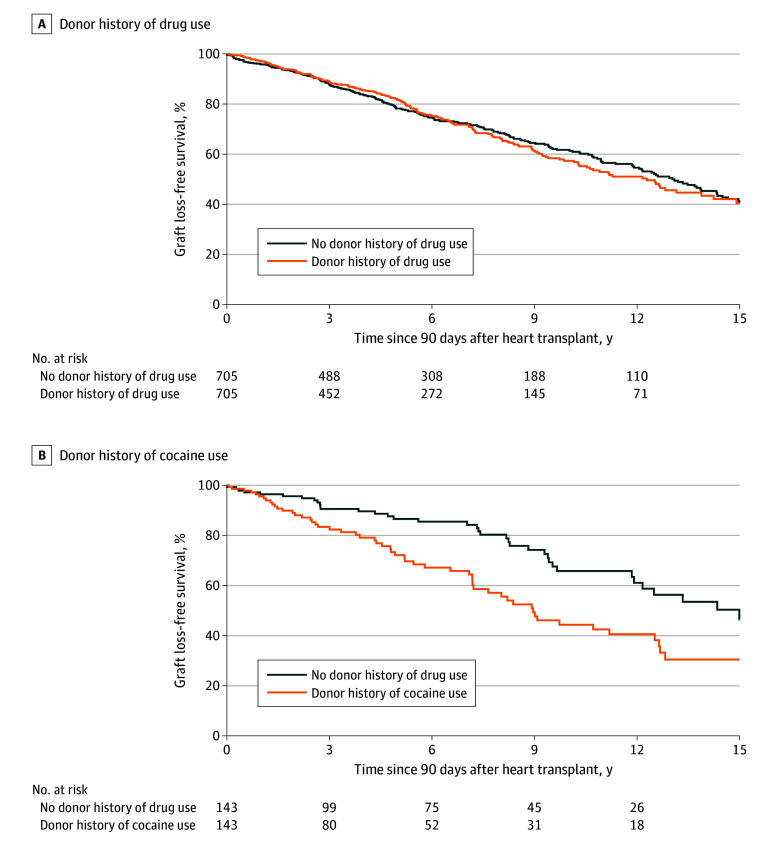
Long-Term Graft Survival in 90-Day Survivors Who Received a Heart From a Donor With vs Without a History of Drug or Cocaine Use, 2000-2020

Among 90-day PS-matched survivors (n = 705 in each group), 204 (28.9% [171 deaths and 33 retransplants]) in the exposure group reached the primary end point compared with 233 (33.1% [192 deaths and 41 retransplants]) in the control group. There was no significant difference in graft survival between groups conditional on surviving 90 days post transplant (Figure 2A). In Cox proportional hazards regression analysis accounting for PS matching, there was no association of donor HDU with long-term survival among 90-day survivors (HR, 1.04 [95% CI, 0.87-1.25]; *P* = .68). Furthermore, there was no association of donor HDU with long-term survival when additionally adjusted for year of transplant, recipient age, and donor age (HR, 1.09 [95% CI, 0.90-1.32]; *P* = .38). These findings were similar when the analysis was limited to matched pairs who received a heart from a donor who had used drugs within 6 months before transplant (HR, 1.02 [95% CI, 0.81-1.29]; *P* = .86).

A total of 143 children who received a heart from a donor with a history of cocaine use were analyzed after 90 days post transplant. Of these children, 56 (39.2% [46 deaths and 10 retransplants]) reached the primary end point compared with 37 (25.9% [26 deaths and 11 retransplants]) in the PS-matched control group. Figure 2B compares long-term graft survival in children who received a heart from a donor with a history of cocaine use vs the control group. The probability of 5-year graft survival was 0.72 (95% CI, 0.62-0.80) in children who received a heart from a donor with a history of cocaine use vs 0.87 (95% CI, 0.79-0.92) in the control group. The probability of 10-year graft survival was 0.44 (95% CI, 0.33-0.55) and 0.66 (95% CI, 0.54-0.75) in the 2 groups, respectively. In Cox proportional hazards regression analysis accounting for PS matching, children who received a heart from a donor with a history of cocaine use had significantly higher risk of graft loss compared with those in the control group (HR, 2.03 [95% CI, 1.35-3.06]; *P* = .001). This association remained significant in a Cox proportional hazards regression model that additionally adjusted for year of transplant, recipient age, and donor age (HR, 1.83 [95% CI, 1.20-2.79]; *P* = .005). Older donor age was also associated with a higher risk of graft loss.

### Additional Analyses

**Figure 3.**
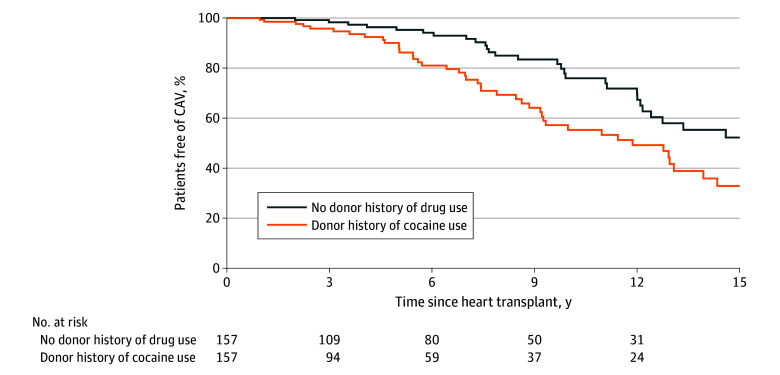
Freedom From Coronary Artery Vasculopathy (CAV) in Pediatric Patients Who Received a Heart From a Donor With a History of Cocaine Use vs Without a History of Drug Use, 2000-2020

Figure 3 illustrates a significantly shorter time to diagnosis of coronary artery vasculopathy in 157 children who received a heart from a donor with a history of cocaine use vs the PS-matched control group (*P* = .005, log-rank test). Of these 157 children, 53 (33.8%) died after transplant. Centers assigned 1 of 25 codes as the cause of death. Death was related to immune suppression (acute rejection, acute infection, or malignancy) in 10 patients. Among the 157 PS-matched children in the control group, there were 36 deaths (22.9%); 6 were related to immune suppression.

## Discussion

The drug use epidemic in the US continues to cause many premature deaths in young adults. The availability of organs from donors with HDU has benefited adults and older children awaiting organ transplant in the US with a decline in median waitlist times.^1^ In this cohort study, we assessed the association of using hearts from donors with HDU with graft survival in pediatric HT recipients. The study has 3 main findings: First, we found a progressive increase in the proportion of donors with HDU over time, as described in adult HT recipients.^2^ Second, receiving a heart from a donor with HDU was not associated with a higher risk of 90-day graft loss. Third, among children who survived 90 days post transplant, donor HDU was associated with higher risk of long-term graft loss only if the donor had a history of cocaine use. These findings may be clinically important when evaluating donors with HDU for pediatric HT candidates.

Analyses of adult HT patients have found no association of receipt of a heart from a donor with HDU with posttransplant 1-year or longer-term survival.^2,3^ Even donor use of cocaine was not associated with worse outcomes, with 10-year HT survival of 60%.^11,12^ Similar results were described in adult recipients in an International Thoracic Transplant Registry report with a focus on donor characteristics.^4^ In pediatric recipients, the registry report noted the lack of association of 1-year posttransplant survival with donor HDU (cocaine or other drugs); however, 5-year survival in children who received a heart from a donor with HDU was worse.^5^ Because these analyses were univariate comparisons, the potential confounding from transplant era and older age of donors with HDU were not accounted for. In this study, we included recipient and donor variables as well as era of transplant in the PS model, thereby minimizing confounding by variables associated with a donor with HDU. We further adjusted for donor and recipient age and year of transplant to reduce their confounding effect. This approach confirmed the association of donor cocaine use with HT outcomes, which was more in line with the conventional wisdom of exercising caution in considering such donors for pediatric HT candidates.

Donor cocaine exposure is of interest, given its known potential for acute and chronic cardiotoxicity and risk of coronary vasospasm and thrombosis.^11,12,13^ Chronic exposure disrupts endothelial activity, may accelerate atherosclerosis, and may lead to left ventricular hypertrophy, decreased end-diastolic volume, and increased filling pressures.^13,14^ Long-term cocaine users are at increased risk of diastolic heart failure.^15^ These effects of cocaine in a donor heart could lead to accelerated coronary artery disease and diastolic failure in a HT recipient.^14,15^ Focal ischemia could also become a nidus for arrhythmia.^16^ Our analysis showed a shorter time to coronary artery vasculopathy in pediatric patients who received a heart from a donor with a history of cocaine use compared with the control group. Because the mechanism of death is difficult to ascertain in registry data, we are unable to determine causal pathways for increased risk of graft loss, however. Why donor cocaine use is not associated with outcomes in adult HT recipients is also unclear. These differences from children may be due to more experience in assessing such offers by adult HT teams, such as by requesting heart catheterization and a coronary angiogram prior to acceptance. They may also be due to shorter graft survival in adult compared with pediatric HT recipients.^15^ Detecting harm from donor cocaine exposure in adult HT recipients may be more challenging, given a larger attrition rate from other transplant-related causes in adults, which may mask the deleterious effects of cocaine exposure.

### Study Implications

The findings of this study suggest that hearts from donors with HDU, including those with a history of cocaine use, are effective in acute salvage of wait-listed pediatric candidates. This also applies to long-term outcomes for recipients of HT from donors with HDU other than cocaine. However, the association of donor history of cocaine use with risk of long-term graft loss appears clinically important. The relative risk of graft loss, the temporal relationship between exposure and outcome, the physiologic basis of the association, and the adequacy of adjustment for measured confounders in the current analysis all suggest a causal role. This association is impractical to be studied in a randomized clinical trial. Therefore, the findings should be considered important when assessing a donor for pediatric HT candidates. Specifically, the significantly lower probability of 5-year and 10-year graft survival in children who receive a heart from a donor with a history of cocaine use must be weighed against the competing risks of waitlist morbidity and mortality, sensitization against a given donor, and the likelihood of additional offers.

### Limitations

This study has some limitations. This was a retrospective study using registry data, with inherent limitations of such data. However, UNOS (being responsible for US transplant allocation) mandates submission of these data from all transplant centers. The data are audited periodically and are used to generate center-specific reports. Second, except for donor use of cocaine, there was no further distinction among other drugs used. Furthermore, the details of frequency, degree, dominant drug, and multiple drugs were not available. Third, the number of patients who received a heart from a donor with a history of cocaine use was small; this would normally result in false-negative results (type II error) but can also occasionally result in false-positive studies.^17,18^ Although this appears unlikely considering the relative risk of graft loss in those who received a heart from a donor with a history of cocaine use, the results should be confirmed in other cohorts.

## Conclusions

In this cohort study of US pediatric patients who received HT between 2000 and 2020, there was no association of posttransplant 90-day graft survival with receiving a heart from a donor with HDU; however, donor cocaine use was associated with a statistically significant and clinically important higher risk of long-term graft loss. These findings may be important to consider when assessing donors with HDU for pediatric HT candidates.

## Notes

Supplement.
Data Sharing Statement
