# Supplementary material for: Donor History of Drug Use and Graft Survival in Pediatric Heart Transplant Recipients
Source: JAMA Netw Open. 2025 Apr 28;8(4):e257766. doi: 10.1001/jamanetworkopen.2025.7766 (PMC12038511; doi:10.1001/jamanetworkopen.2025.7766)
Supplement: Supplement. — Data Sharing Statement [file jamanetwopen-e257766-s001.pdf]

Data Sharing Statement

Esteso. Donor History of Drug Use and Graft Survival in Pediatric Heart Transplant Recipients. *JAMA Netw Open*. Published April 28, 2025. doi:10.1001/jamanetworkopen.2025.7766

Data
Data available: No

Additional Information
Explanation for why data not available: These data are available to all investigators from UNOS after paying a nominal fee and signing a data use agreement that includes a provision to not share data.
